# The Effects of Tooth Brushing on Whole Salivary Flow Rate in Older Adults

**DOI:** 10.1155/2018/3904139

**Published:** 2018-02-26

**Authors:** R. H. Affoo, K. Trottier, R. Garrick, T. Mascarenhas, Y. Jang, R. E. Martin

**Affiliations:** ^1^Department of Communication Sciences & Disorders, College of Health Professions, Central Michigan University, Mount Pleasant, MI, USA; ^2^Graduate Program of Health and Rehabilitation Sciences, Western University, London, ON, Canada; ^3^Department of Psychology, Western University, London, ON, Canada; ^4^Department of Physiology & Pharmacology, Western University, London, ON, Canada; ^5^School of Communication Sciences & Disorders, Elborn College, Western University, London, ON, Canada; ^6^Department of Physical Medicine & Rehabilitation, Schulich School of Medicine & Dentistry, Western University, London, ON, Canada; ^7^Department of Dentistry, Schulich School of Medicine & Dentistry, Western University, London, ON, Canada; ^8^Faculty of Health Sciences, Western University, London, ON, Canada

## Abstract

**Objectives:**

(1) To determine whether manual (MTB), or electric, tooth brushing (ETB) modulates whole salivary flow rate in older adults who are free of systemic disease. (2) To determine the duration of the brushing-related modulation of salivary flow rate. (3) To compare salivary flow rate modulation associated with MTB and ETB.

**Method:**

Twenty-one adults aged 60 years and older participated in two experimental sessions during which they used a manual, or electric, toothbrush to brush their teeth, tongue, and palate. Whole salivary flow rates were determined using the draining method before, during, and after brushing. Differences in salivary flow rates across time periods, and between conditions, were examined using paired samples *t*-tests applying a Holm-Bonferroni sequential procedure (*p*_corr_ < 0.0045). The relationship between tooth brushing and age with respect to maximum salivary flow rate increase was examined using Pearson's correlation coefficient (*p* < 0.05).

**Results/Conclusion:**

Whole salivary flow rates increased during, and for up to 5 minutes following, tooth brushing in adults aged 60 years and older who were free of systemic disease. The salivary effects of MTB and ETB were not significantly different. A moderate, positive correlation was observed between tooth-brushing-related maximum salivary flow rate increase and age.

## 1. Introduction

The oral tissues are among the most richly innervated of any in the human body. Sensory receptors in the tongue, periodontal ligament, gingiva, and palate convey an extensive range of sensory information including pressure, stretch, vibration, pain, and temperature [1–3]. These forms of sensory stimulation are thought to modulate salivary flow rate [4–6].

Tooth brushing has the capacity to produce pressure, stretch, and vibratory mechanical stimulation on the tongue, periodontal ligament (through pressure on the teeth), gingiva, and palate. Thus, tooth brushing may be hypothesized to modulate salivary flow rate. Current literature examining tooth brushing as a form of saliva stimulation is limited. Hoek and colleagues [7] identified a transient increase in salivary flow rate in 14 healthy adults during the initial five minutes following manual brushing. Ligtenberg and group [8] reported that, in 80 healthy students, salivary flow rates increased significantly after brushing with water and after brushing with dentifrice, and salivary flow rates remained increased for 60 minutes after brushing. In older adults with clinically significant hyposalivation, Papas et al. [9] reported that an electric toothbrush (ETB) tended to stimulate greater salivary flow rates compared with a manual toothbrush (MTB) for up to 45 minutes after stimulation. Taken together, these studies provide conflicting findings with regard to the duration of increased salivary flow following tooth brushing. Furthermore, salivary responses to tooth brushing in older adults without clinically significant hyposalivation have not been examined. A clearer understanding of the effects of tooth brushing on salivary flow rate would inform consideration of the feasibility of this form of stimulation as an oral health intervention.

Therefore, the present study (i) examined whether manual, or electric, brushing of the teeth, tongue, and palate modulates whole salivary flow rate in older adults free of major systemic disease, (ii) determined the duration of brushing-related modulation of salivary flow rate, and (iii) compared the salivary flow rate modulation associated with manual and electric tooth brushing. We hypothesized that tooth brushing would increase salivary flow rate during, and for 5 to 30 minutes following, tooth brushing [7–9], with the ETB conferring a larger effect compared with the MTB. Given that older adults have a lower unstimulated whole salivary flow rate than younger adults [10], we further hypothesized that the maximum salivary flow rate increase in response to tooth brushing would decrease with increasing participant age. A version of this study has been published in thesis [11] and abstract form [12].

## 2. Methodology

Twenty-one nonsmoking adults who were free of major systemic disease volunteered as participants. Candidates were excluded if they had fewer than 20 natural teeth, complained of xerostomia, or had been seen by a dentist in the seven days immediately prior to the experimental session. The sample size was based on a power calculation indicating that a sample of *N* = 20 was sufficient to detect a difference of one standard deviation (*d* = 1.0) in a two-level, within-subjects independent variable 80.8% of the time, using a 0.01 alpha level and assuming a within-subjects correlation of 0.30.

Relevant participant data relating to age, medical history, and dental history were collected during a brief interview prior to the experimental session. Participants were instructed to eat breakfast and complete their morning oral hygiene routine by 0800 and to refrain from eating or drinking prior to the study session. Each session commenced at 0900 and lasted approximately 120 minutes. Each subject gave written informed consent before participating in the study, which was approved by the Western University Research Ethics Board for health sciences research involving human subjects.

Each person participated in two experimental sessions. Participants were randomly assigned to one of two groups. Group one used a MTB in the first experimental session and an ETB during the second experimental session. Group two followed the same procedure in reverse order. The two experimental sessions were separated by at least one day, and no more than 21 days, across subjects.

A visual inspection of the oral cavity was completed to rule out gross anatomic abnormalities and to ensure each participant had at least 20 natural teeth. Participants then rinsed their mouth with distilled water.

Participants were seated in a chair that was stationed in front of a table. Transducers were positioned on the participant: belt-mounted movement sensors positioned around the participant's neck (Model 1585, CT2 Pediatric Piezo Respiratory Effort Sensor, Pro-Tech Services, Inc., License Number 69444) and upper abdomen (Model 1582, CT2 Adult Piezo Respiratory Effort Sensor, Pro-Tech Services, Inc., License Number 69444) recorded neck and respiratory movements, respectively. An omnidirectional electret microphone (F-SM Snore Electret Microphone, Pro-Tech Services, Inc., License Number 69446), affixed to the participant's neck with medical tape, monitored the acoustic signal from the upper airway through the tissues of the neck. These physiologic signals were recorded continuously throughout the session using an AS40 Comet Series PSG/EEG Portable System (Astro-Med Inc., License Number 65827). In addition to these physiologic signals, a lateral-plane video recording of the participant that included the head, neck, shoulders, and chest enabled researchers to observe whether participants swallowed during the saliva collection periods.

**Figure 1:**
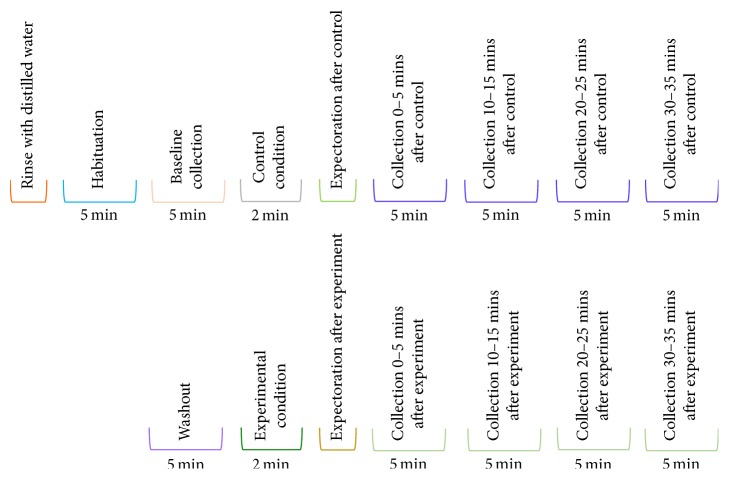
Experimental protocol.

Each of the two study sessions was comprised of a habituation period, control condition, experimental condition, washout period, and eleven salivary collection periods (Figure 1). During the five-minute habituation period, the participant sat at rest and was instructed to minimize their orofacial movements, and video recordings and neck movement, respiratory, and acoustic data were collected. Participants provided saliva samples, using the draining method, into a preweighed beaker [13]. A five-minute saliva-draining collection was performed at baseline following the habituation period and at 0 to 5 minutes, 10 to 15 minutes, 20 to 25 minutes, and 30 to 35 minutes following the control and experimental conditions. The participants were instructed not to swallow their saliva during the draining period. Following the study, the video and physiologic signals were reviewed by RHA for evidence of swallow-related respiratory and laryngeal movement patterns to verify that swallowing had not occurred.

Participants completed the control condition by placing either a Colgate Sensitive Pro-Relief MTB or a Colgate Sonic Power ETB, in the oral cavity (without dentifrice, bristles touching the superior surface of the tongue) and holding it stationary for two minutes. The experimental stimulation condition involved the participant actively brushing their teeth, tongue, and palate (without dentifrice) for two minutes. Participants were instructed not to swallow their saliva during the control and experimental tooth-brushing conditions. Immediately following both conditions, participants expectorated their saliva into a preweighed beaker. These salivary collections will be referred to as the salivary flow rates collected “during” the control and experiment. Participants sat for a five-minute washout period between the control condition and the experimental intervention.

Participants were trained to use a standardized tooth-brushing protocol when completing each two-minute oral cavity stimulation intervention. For the MTB intervention, participants were instructed to use a modified Bass technique [14] and systematically brush the outer, inner, and chewing surfaces of the teeth, as well as the tongue and palate. Similar instructions were used for the ETB intervention. The instructions for each of the standardized tooth-brushing protocols were presented in a video prior to the experimental intervention.

Subject data relating to age, medical history, and dental history were analyzed by RHA.

All beakers were weighed immediately prior to the experiment and immediately following each saliva-draining period.

Whole salivary flow rates were calculated for each collection period in g/min. Planned contrasts were completed using paired samples *t*-tests and a Holm-Bonferroni sequential procedure to correct for familywise error (*p*_corr_ < 0.0045) [15]. The comparisons of interest included (i) baseline rates compared with the flow rates during the control and experimental conditions and (ii) baseline rates compared with the flow rates from 0 to 5 minutes, 10 to 15 minutes, 20 to 25 minutes, and 30 to 35 minutes after the control and experimental conditions for both the MTB and ETB protocols. The effects of the control and treatment conditions on salivary flow rate were estimated using Cohen's *d* [16].

Differences between manual and electric tooth-brushing effects on salivary flow rate were examined through descriptive comparisons of the effect sizes (Cohen's *d*) calculated for the MTB and ETB conditions. Additionally, the maximum salivary flow rate increases (i.e., the difference between salivary flow rates during tooth brushing and baseline) associated with the MTB and ETB were compared using a paired samples *t*-test (*p* < 0.05). The relationship between the maximum salivary flow rate increases associated with the manual and electric tooth brushing was examined using a Pearson's correlation coefficient (*p* < 0.05).

The relationship between age and the maximum salivary flow rate change was examined using Pearson's correlation coefficient (*p* < 0.05). Medication use was explored using descriptive statistics and a chi-square test (*p* < 0.05).

Statistical analyses were completed using SPSS [17] and Microsoft Excel.

## 3. Results

### 3.1. Participants

Twenty-one adults participated in the study (62–83 years of age, M = 71.33 years, SD = 6.46 years; 11 female). All participants had at least 20 natural teeth (range = 22–28, M = 25.67, SD = 1.93). No participants were observed to swallow during the salivary collection periods. Seven of the 21 participants reported taking no medications with potential xerogenic effects and 14 of the 21 participants reported taking xerogenic medications (range = 0–4, M = 1.25, SD = 1.11) [18]. The results of a chi-square test (*χ*^2^ = 3.19, *p* = 0.36) indicated that the numbers of participants taking zero, one, two, or three or more medications were not significantly different (data not shown). Xerogenic medications used by the participants included antihyperlipidemic, antiulcer, antihypertensive, and anti-inflammatory agents.

### 3.2. Effects of Tooth Brushing on Salivary Flow Rate

**Figure 2:**
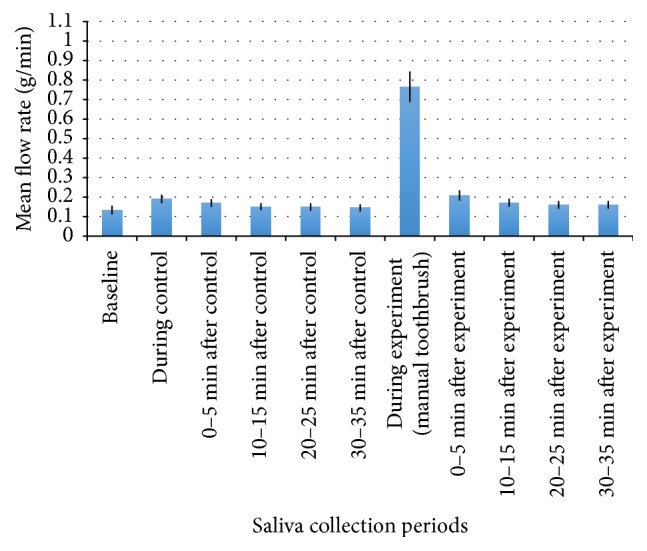
Mean whole salivary flow rate collected before, during, and after control and manual tooth brushing. Mean flow rate (g/min) is represented by the bars and the error bars indicate SE.

**Figure 3:**
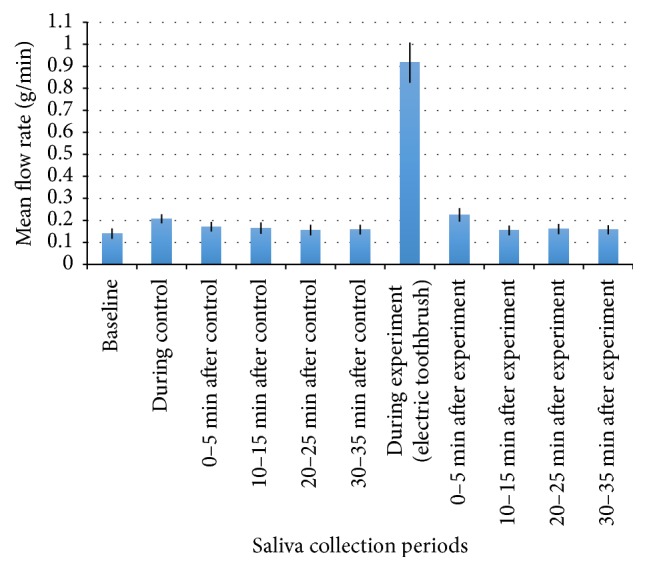
Mean whole salivary flow rate collected before, during, and after control and electric tooth brushing. Mean flow rate (g/min) is represented by the bars and the error bars indicate SE.

The mean whole salivary flow rates for each collection period are presented in Figure 2 (MTB) and Figure 3 (ETB).

Planned contrasts were completed using paired samples *t*-tests. The Holm-Bonferroni sequential procedure was used to correct for familywise error (*p*_corr_ < 0.0045) (Table 1).

A significant, large increase (*d* = 2.50) in salivary flow rate was observed during manual tooth brushing compared with the baseline salivary flow rate (M = 0.63, SD = 0.34, *p* < 0.0045) and with the control condition salivary flow rate (M = 0.58, SD = 0.33, *p* < 0.005). The salivary flow rate collected from 0 to 5 minutes after manual tooth brushing was significantly, moderately increased (*d* = 0.661) compared with the baseline salivary flow rate (M = 0.07, SD = 0.07, *p* < 0.0055).

A significant, moderate increase (*d* = 0.672) in salivary flow rate was observed during the control condition compared with the baseline salivary flow rate during the electric toothbrush protocol (M = 0.07, SD = 0.08, *p* < 0.006).

A significant, large increase (*d* = 2.54) in salivary flow rate was observed during the electric tooth brushing compared with the baseline salivary flow rate (M = 0.78, SD = 0.37, *p* < 0.0045) and with the control condition salivary flow rate (M = 0.71, SD = 0.35, *p* < 0.005). The salivary flow rate collected from 0 to 5 minutes after electric tooth brushing was significantly, moderately increased (*d* = 0.681) compared with the baseline salivary flow rate (M = 0.08, SD = 0.08, *p* < 0.0055).

### 3.3. Manual Compared to Electric Tooth Brushing

The effect sizes associated with the salivary flow rate collected during manual and electric tooth brushing were similar (MTB: *d* = 2.40; ETB: *d* = 2.54). The maximum salivary flow rate increases associated with the MTB and ETB were not significantly different (M = 0.15, SD = 0.42, *p* = 0.129). A small correlation between the maximum salivary rate increases associated with the MTB and ETB protocols (*r*(19) = 0.30, *p* = 0.184) was identified.

### 3.4. Age and Salivary Flow Rate

Baseline salivary flow rate data for the MTB protocol were not normally distributed, as assessed by Shapiro-Wilk's test (*p* = 0.001). Baseline data for the ETB protocol, however, were normally distributed (*p* > 0.05). A Spearman rank-order correlation was completed using the MTB data and a Pearson's correlation coefficient was completed with the ETB data. Age was not significantly correlated with the baseline salivary flow rate for the MTB (*r*_*s*_(19) = 0.05, *p* = 0.84) or ETB protocols (*r*(19) = 0.1, *p* = 0.68).

**Figure 4:**
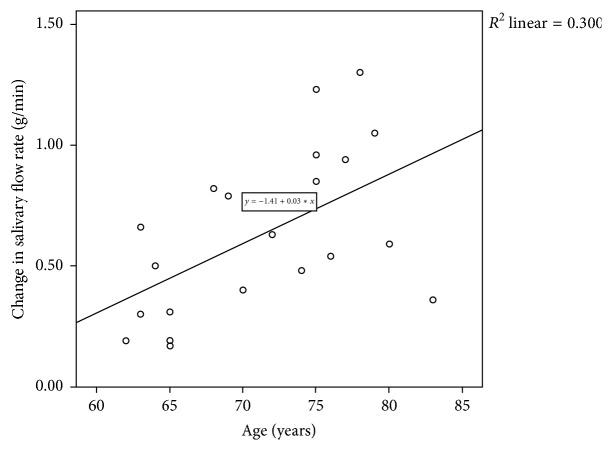
Scatterplot illustrating the correlation between age (in years) and the maximum salivary increase (g/min) associated with manual tooth brushing.

Age was moderately correlated with the maximum salivary flow rate increase (*r*(19) = 0.55, *p* = 0.01) for the MTB condition (Figure 4). A small, nonsignificant correlation was observed (*r*(19) = 0.18, *p* = 0.44) for the ETB condition.

## 4. Discussion

The results of the current study suggest that manual or electric tooth brushing is associated with short-duration increase in whole salivary flow rate in healthy older adults. Whole salivary flow rates increased significantly for up to five minutes following either manual or electric brushing of the teeth, tongue, and palate. The increase in salivary flow rate immediately following the two-minute brushing period was large whereas the increase at five minutes following the brushing period was moderate. The present study also found that holding a deactivated ETB in a stationary position in the oral cavity resulted in a transient increase in whole salivary flow rate.

These results are similar to those of Hoek et al. [7] who reported that tooth brushing induced a transient increase in saliva flow rate during the following five minutes. Our results are not consistent with those of Ligtenberg et al. [8], however, who reported that, after brushing with water or dentifrice, the salivary secretion rate increased significantly for 60 minutes. This inconsistency may be due to the additional oral stimulation associated with brushing with water or dentifrice which could have caused gustatory or thermal stimulation in addition to the mechanical stimulation associated with tooth brushing, potentially influencing the duration of increased salivary flow rates following stimulation.

We hypothesized that electric tooth brushing would have a larger effect on salivary flow rate compared with manual tooth brushing. Papas et al. [9] reported that Sonicare ETB users tended to have increased salivary flow rates after stimulation compared with MTB users. In contrast, Hiraba et al. [6] found that increasing the frequency of vibratory stimulation applied to the facial skin overlying the belly of the masseter muscles did not result in greater salivation, possibly because individual mechanoreceptors differ in their threshold sensitivity to vibration. We found no significant differences between the two tooth-brushing protocols with regard to increasing whole salivary flow rates which suggests that the increased vibration associated with ETBs may not result in a greater salivary response compared with MTBs.

In our post hoc analysis, we examined the relationship between age and the maximum salivary flow rate increase (i.e., the maximum difference between salivary flow rates during baseline and tooth-brushing conditions). We hypothesized that, as age increased, maximum salivary flow rate increase would decrease. This hypothesis, however, was not supported by the results. Instead, a moderate, positive correlation was observed between age and maximum salivary flow rate increase. That is, as age increased, so too did the maximum salivary flow rate increase observed during tooth brushing. In order to explain this phenomenon, we hypothesized that the older participants had reduced baseline salivary flow rates compared with the relatively younger participants. A lower baseline salivary flow rate might increase the potential for response to stimulation, resulting in a stimulated salivary flow rate similar to the relatively younger participants. When we then explored the relationship between age and baseline salivary flow rates, however, no significant correlations were identified. The oldest adults in our sample demonstrated a more robust salivary response to tooth brushing compared with the relatively younger subjects. Although parotid and minor salivary flow rates do not decline with increasing age [10] and aspects of somatosensation also do not decline with advancing age [19, 20], this evidence alone does not explain why we observed a positive relationship between age and maximum salivary flow rate increase. Older subjects were not observed to have more teeth compared with the younger subjects, younger subjects were not observed to be taking more xerogenic medications compared with the older subjects, and the males and females were equally distributed with regard to age (data not shown).

The positive relationship between age and maximum salivary flow rate increase was found to be moderate for manual tooth brushing but small for electric tooth brushing. This finding is provocative given that comparison of the salivary flow rate increases associated with the two types of brushing revealed no statistically significant differences. We examined the relationship between maximum salivary flow rate increases associated with the MTB and ETB and identified only a small correlation.

### 4.1. Limitations

Epithelial cells are continually being shed from the oral mucosa into saliva and it has been estimated that the surface cells stay attached for only about three hours before being desquamated [21]. Participants in our study reported completing their early morning oral hygiene routine at least one hour prior to the experiment and all participants rinsed their oral cavities with distilled water immediately prior to participating. The elements of the present experimental protocol reduce the likelihood of epithelial cells making a significant contribution to the salivary collection and adding to the weight of the saliva samples. Nevertheless, it is possible that epithelial cells as well as plaque and residual food debris in the mouth, displaced by tooth brushing, may have contributed to the weight of the saliva samples.

One limitation of this study is that we did not complete a comprehensive dental exam on participants prior to study enrollment. We do not therefore have detailed information regarding the periodontal status of our participants.

Participants were mainly recruited from an exercise program, introducing a potential bias in that the participants may have been more health-conscious than the general population.

The ETB used in the experiment had a brush head shaped similarly to the MTB and was not circular in shape. A circular brush is a popular shape among name brand ETBs. Therefore, we may not have employed a representative ETB.

Glandular saliva was not collected in the present study. Thus, it is unclear which glands contributed more saliva to the increased flow rates in response to the tooth brushing. Based on previous work in this area, however, showing that the percentage contribution from the parotid gland increases to more than 50% of total salivary secretions during stimulation [22–24], we would predict that the parotid glands contributed the greatest percentage of saliva to the increased flow rates observed.

### 4.2. Clinical Implications

The present study showed a significant increase in whole salivary flow rate during, and for up to five minutes following, tooth brushing in older adults. Although the salivary flow rate modulation was transient, this increase in salivary flow would be expected to contribute to reducing oral bacterial load and increasing oral lubrication.

Increasing salivary volume may affect salivary clearance in that the volume of oral saliva contributes to triggering of the pharyngeal stage of swallowing [25] and increasing swallowing rate [26]. The act of swallowing secreted saliva reduces the concentration of exogenous substances in the oral cavity and is beneficial for oral health [27]. Thus, an increase in salivary volume would be expected to stimulate increased salivary clearance in an individual who has potentially harmful substances in the oral cavity, with beneficial effects on oral and overall health.

The correlation between age and maximum salivary flow rate increase indicates that the older adults in the present study experienced greater salivary responses to tooth brushing compared with the relatively younger participants. Given that aging is associated with reduced salivary flow [10], and that reduced salivary flow may lead to impaired oral health [28], older adults are at greater risk of developing poor oral health. Therefore, the capacity to increase salivary secretions among older individuals has important clinical implications with regard to improving the oral health of this cohort.

## 5. Conclusion

The present study suggests that tooth brushing stimulates saliva production for up to five minutes in adults aged 60 years and older who are free of systemic disease. Older participants had a more robust salivary response to tooth brushing compared with younger participants, suggesting that older adults may particularly benefit from tooth brushing to stimulate salivary secretion.

## Figures and Tables

**Table 1:** Salivary flow rate comparisons and the Holm-adjusted *p* values.

Comparison	Mean difference	Holm-adjusted *p*
*Manual toothbrush*		
Baseline, during brushing	0.63	0.0045
During control, during brushing	0.58	0.0050
Baseline, 0 to 5 minutes after brushing	0.07	0.0055
*Electric toothbrush*		
Baseline, during brushing	0.78	0.0045
During control, during brushing	0.71	0.0050
Baseline, 0 to 5 minutes after brushing	0.08	0.0055
Baseline, during control	0.07	0.0060
